# Pleiotropic Functions and Biological Potentials of Silver Nanoparticles Synthesized by an Endophytic Fungus

**DOI:** 10.3389/fbioe.2020.00095

**Published:** 2020-02-21

**Authors:** Radhika Chandankere, Jayabaskaran Chelliah, Kamalraj Subban, Vanitha C. Shanadrahalli, Amreesh Parvez, Hossain M. Zabed, Yogesh C. Sharma, Xianghui Qi

**Affiliations:** ^1^School of Food and Biological Engineering, Jiangsu University, Zhenjiang, China; ^2^Department of Biochemistry, Indian Institute of Science, Bengaluru, India; ^3^Department of Chemistry, Indian Institute of Technology, Banaras Hindu University, Varanasi, India

**Keywords:** silver nanoparticles, antithrombin potential, endophytic fungus, biogenic synthesis, *Colletotrichum incarnatum*

## Abstract

In recent years, the biological synthesis of silver nanoparticles (AgNPs) from microorganisms has become an emerging trend for developing biocompatible nanomaterials that finds applications in nano and biomedical sectors. In the present study, we demonstrated a facile, green and eco-friendly method for AgNPs synthesis using the endophytic fungi (*Colletotrichum incarnatum* DM16.3) isolated from medicinal plant *Datura metel* and its *in vitro* antithrombin and cytotoxic activity. At first, biosynthesis of colloidal AgNPs was predicted by visual observation of color change and UV-visible spectra demonstrated specific surface plasmon resonance peak at 420 nm which confirmed the presence of nanoparticles. Microscopic analyses revealed the structure of highly aggregated, spherical and crystalline AgNPs in the diameter range of 5–25 nm. Transform infrared spectroscopy (FT-IR) spectral analysis confirmed the presence of probable biomolecules required for the reduction of silver ions. *In vitro* evaluation of thrombin activity demonstrates that AgNPs could exert strong inhibition against both thrombin activity (87%) and thrombin generation (84%), respectively. Further, *in silico* based mechanistic analysis yielded a better insight in understanding the probable amino acids responsible for AgNPs binding with thrombin protein. Similarly, *in vitro* cytotoxicity of synthesized AgNPs on human epithelial cells using MTT assay did not produce any substantial effects after 24 h exposure which indicates excellent biocompatibility nature, whereas notable toxicity was observed on human cancerous (HeLa) cells at 50 μg/mL (IC_50_ value). In addition, assessment of AgNPs at 10 μg/mL concentration via crystal violet method on biofilm forming Gram-positive (*Vibrio cholerae*) and Gram-negative bacteria (*Bacillus cereus*) revealed inhibition up to 85 and 46%, respectively. Overall, this study showed the possibility of microbially synthesized AgNPs as a potent inhibitor for managing acute thrombosis and highlighted their role for other biomedical applications.

## Introduction

Fungal nanotechnology is an upcoming field of nanotechnology and it has consequently gained tremendous impetus in fabricating nanoparticles with a wide range of applications toward human welfare (Vivian et al., 2018). Metallic nanoparticles (NPs) are being employed in the field of agri-food, diagnostics, therapeutics, and medical device development, with fungal NPs recently used in application ranging from drug development to the pharmaceutical and food industries (Baker et al., 2015). Fungi are the preferred microorganisms as they are fast-growing, easy to handle and produce large quantities proteins needed for the synthesis of NPs. Previously, different types of metal NPs have been biosynthesized namely, titanium, copper, zinc, gold, and silver (Mukherjee et al., 2001; Kirthi et al., 2011; Balakumaran et al., 2015; Jafarirad et al., 2016; Prabhu et al., 2017; Mohamed et al., 2019). Among these NPs, silver nanoparticles (AgNPs) are found to be superior, as they have a larger surface area that results in greater surface energy, catalytic activity, and biochemical reactivity (Syed and Ahmad, 2012; Neethu et al., 2018; Ying et al., 2018; Zhou et al., 2018). Some of the most commonly used fungal genera for the biosynthesis of AgNPs are *Fusarium*, *Trichoderma*, *Cladosporium*, *Aspergillus*, *Penicillium*, and *Phanerochaete* (Bhainsa and D’Souza, 2006; Vahabi et al., 2011; Syed and Ahmad, 2012; Neethu et al., 2018).

Recently, fungal-mediated AgNPs bound with herbal drugs have been found more beneficial and competent over conventional forms of drugs. Amongst fungi, not much work has been done on the fabrication of endophytic fungi from medicinal plants for the synthesis of AgNPs and reports are still limited (Singh et al., 2013). According to the literature, endophytic fungi are a promising source for drug discovery by providing a unique way of fabricating a range of AgNPs, which showed a broad pharmacologic potential (Loo et al., 2018; Naidu et al., 2019). Medicinal plant-derived endophytic fungi have received broad attention due to the unusual living environment, that is exposed to the high temperature and salinity (Vahabi et al., 2011). In this context, we have used *Datura metel* belonging to the Solanaceae family which is a widely distributed herb native to China and India and is described as the divine drug in the traditional medicines used for the treatment of a number of ailments. According to the literature, various endophytic microorganisms were isolated from *D. metel* that have various biological activities, such as anticancer, antioxidant, antimicrobial, anti-inflammatory, and antifungal (Narendra and Uday, 2014; Baker et al., 2015; Dakal et al., 2016; Loo et al., 2018; Majeed et al., 2018; Zhou et al., 2018).

Thrombosis is the formation/presence of a thrombus (blood clot), which hinders the flow of blood in blood vessels, resulting in abnormal coagulation. In other words, this coagulation caused by the bacterial infection is frequently related to the prothrombotic case as it came across the hemostatic abnormalities and further activates the coagulation factors ending up in the formation of unusual clots in the arteries and veins (Kuriakose et al., 2013). In brief, the key reason for the thrombosis in infection is due to the inhibition of fibrinolysis and the generation of tissue factor-mediated thrombin (Xiaowen et al., 2019). Henceforth, antithrombotic drugs (ATPs) are essential to combat these disorders by hindering the thrombosis using thrombin inhibitors drugs. Previously, researchers have reported thrombin inhibitory activity of leaf and flower extracts of *Catharanthus roseus* (Kuriakose et al., 2013). According to the literature, any bioactive compound used for human welfare has to be assessed for its eco-toxicity (Chandankere et al., 2014). Also, it is reported that the plants need to be included to develop a comprehensive toxicity profile for NPs due to its low execution costs (Yin et al., 2012; Zhou et al., 2018). Therefore, based on the potential of AgNPs, the current study also aims to develop an innovative bioactive agent in eco-friendly fungal-based nanomaterials.

As the gamut of the research on various bioactive compounds from endophytic fungi harbored in medicinal plants (Singh et al., 2013; Rasool and Hemalatha, 2017), herein we have selected *D. metel* for isolation of a novel fungal isolate. In the present study, *C. incarnatum* DM16.3 was isolated from the healthy leaves of *D. metel* and exploited for the biosynthesis of AgNPs. The novel finding of the present study is the demonstration of thrombin inhibitory activity of the AgNPs synthesized by *C. incarnatum* with an insight into its molecular simulation dynamics studies. The morphology and structure of the biosynthesized AgNPs were characterized using UV-visible absorption spectroscopy, scanning electron microscopy (SEM), and transform infrared spectroscopy (FT-IR). Subsequently, experiments were conducted for *in vitro* antibiofilm studies against the human pathogens and cytotoxic assay toward normal and cancerous cells. In addition, phytotoxicity of AgNPs was evaluated against the seeds of two model bio-indicator plants, namely *Cucumis sativus* and *Vigna radiata* to reveals the eco-toxicity of AgNPs.

## Materials and Methods

### Chemicals and Biological Strains

Silver nitrate (AgNO_3_) was purchased from HiMedia Laboratories Pvt. Ltd., Mumbai, India. All other fine chemicals were obtained from Sigma-Aldrich (HiMedia). The pathogenic indicator strains used in this study were Gram-negative bacteria (*Vibrio cholerae*) and Gram-positive bacteria (*Bacillus cereus*) that were procured from the Microbial Type Culture Collection, Chandigarh, India. Both strains were cultured in Luria Broth (HiMedia) at 37°C under aerobic conditions. All the cultures were stored at −80°C in broth supplemented with 20% glycerol.

### Plant Material and Isolation of Endophytic Fungus

Healthy leaves of *D. metel* were collected from the nursery of the Indian Institute of Science campus, Bengaluru, India. The leaves were washed thoroughly with running tap water to remove epiphytic fungi. The leaf samples were then rinsed with sterile distilled water and treated with 20% commercial bleach supplemented with 0.1% Tween-20 for 5 min, followed by rinsing with sterile distilled water. The samples were soaked for 20 min in 100 mL solution consisted of (in 100 mL) fungicide; Bavistin (30 mg), Tetracycline (0.6 mg), and Rifampicin (0.6 mg) supplemented with 0.1% Tween-20. After treatment, samples were rinsed with sterile distilled water. The surface-sterilized samples were cut into small segments (1–2 mm) aseptically. All the sterile segments were evenly placed on Petri dishes containing potato dextrose agar (PDA) medium supplemented with 100 mg/L ampicillin and incubated at 25 ± 2°C for 7 days. The Petri dishes were observed at regular intervals for the fungal growth and mycelia emerged from the surface of the leaf segments were transferred on to the fresh PDA plates, and incubated for 48 h.

### Morphological and Identification of Endophytic Fungal Isolated

Photomicrographs of conidiogenous cells and conidia of the isolated fungal strain were taken by the phase-contrast microscope fitted with a camera by using a Zeiss light microscope (Olympus BX43). The mycelial samples, conidiogenous cells, and young and mature conidia were suspended in sterile distilled water and observed under 400×.

The fungal genomic DNA was isolated by sodium dodecyl sulfate (SDS) method as described previously (Das et al., 2018). Briefly, the strain was inoculated in the shake flask containing 250 mL PDA medium and incubated at 25 ± 2°C for 5 days. The mycelium was harvested by filtration through cheese cloth and blot dried. Two grams of mycelium were weighed and ground in liquid nitrogen using sterile mortar and pestle. The powdered samples were thoroughly mixed with 4 mL of extraction buffer [1 M Tris–HCl (pH 8.0), 0.5 M EDTA (pH 8.0), 10% SDS, and 5 M NaCl], 3 μL RNase A, and 10 μL ß-mercaptoethanol, and incubated at 60°C for 1 h. subsequently, an equal volume of Tris–HCl (pH 8.0): water-saturated (2:2) phenol was added, mixed well and centrifuged at 6000 × *g* for 5 min. The supernatant was removed and pellets were suspended in the equal volume of chloroform: isoamyl alcohol mixture (24:1) and centrifuged at 6000 × *g* for 5 min. Later, the upper aqueous layer was mixed thoroughly with 0.1 vol of 3 M ammonium acetate and 2 vol of 100% ice-cold ethanol. The solution was incubated at -20°C overnight and centrifuged at 12,000 × *g* for 15 min and the supernatant was discarded. Then the pellet was washed with 70% ice-cold ethanol by centrifugation at 7500 × *g* for 5 min, air-dried and re-suspended in 100 μL of TE buffer/Sterile distilled water. DNA purity was ascertained from the A_260_/A_280_ absorbance ratios. A master mix was prepared using the 10 × Taq buffer, 10 mM dNTPs, Taq polymerase and pipetted into each PCR tubes along with 2 μL of primer (1 μL of forward and 1 μL of reverse primer) and 1 μL of purified DNA extract was added and the total concentration of each tube was made up to 25 μL using sterilized water.

To amplify the internal transcribed spacer (ITS) regions of the fungal endophyte, ITS 1 and 2 primers were used. ITS 1 (5′-TCCGTAGGTGAACCTGCGG-3′) and ITS 2 (5′-GCTGCGTTCTTCATCGATGC-3′) primers amplified the predicted ∼300-bp fragment. DNA was amplified in 25 μL of the reaction mixture in PCR tubes and the PCR tubes were given a small spin and were placed in a PCR thermocycler (MinicyclerTM, Germany) and programmed as follows: the thermal cycle consisted of initial denaturation at 94°C for 6 min, denaturation at 94°C for 50 s, annealing at 54°C for 50 s, extension at 72°C for 50 s, followed by 35 cycles and a final extension at 72°C for 10 min. The amplified DNA fragments were analyzed by agarose gel electrophoresis. Purification of the PCR product was done by GeneJETTM Gel Extraction kit, Fermentas and then send for sequencing at MWG Biotech Ltd., Bangalore, India.

### Biosynthesis of Silver Nanoparticles

For the biosynthesis of AgNPs, *C. incarnatum* DM16.3 was grown in 250 mL Erlenmeyer flasks containing 100 mL of PDA broth medium (pH 7.0, adjusted by 10% Na_2_CO_3_). The culture was incubated on a rotary (120 × *g*) shaker at 25 ± 2°C for 96 h. After incubation, the mycelia were harvested by filtering through Whatman filter paper No. 1 and washed thrice with sterile distilled water to remove any medium component. 10 g of harvested wet mycelial mass was then re-suspended in 100 mL of 1 mM aqueous solution of AgNO_3_ in 250 mL Erlenmeyer flasks followed by incubation at the same condition mentioned above. The mycelium biomass was harvested after complete incubation by filtering through Whatman filter paper No. 1. This was followed by repeated washing with distilled water to remove any medium component from the biomass. Then, 10 g of harvested mycelium was re-suspended in 100 mL of 1 mM aqueous solution of AgNO_3_ solution (prepared in deionized water) and incubated at same condition mentioned above. Concurrently, a positive control of incubating the fungus mycelium with deionized water was also maintained. After the incubation, the cell filtrate (CF) was obtained by passing it through Whatman filter paper No. 1. Later, the CF containing AgNPs was repeated centrifugation at 8000 × *g* for 10 min and the pellets of AgNPs were re-dispersed into pure acetone. After air-drying of the purified AgNPs, they were stored at 4°C for further analysis.

### Characterization of Silver Nanoparticles

Preliminary confirmation of AgNPs formed in the mycelium free fungal filtrate was done through visual observation of color change. The time-dependent formation of AgNPs was observed using a UV-visible spectrophotometer. Biosynthesized AgNPs were confirmed by sampling the reaction solution at regular intervals and the absorption spectra were scanned at a resolution of 1 nm between the wavelength of 300 and 700 nm in a spectrophotometer (SPECORD S-600, Analytik Jena, Germany).

For electron microscopy analysis, the fine powder of AgNPs was dispersed in ethanol on a carbon-coated copper grid. The grid was removed after 10 min and air-dried. The images of AgNPs were taken using transmission electron microscopy (TEM) assisted with energy-dispersive X-ray spectroscopy (EDX) (FEI Tecnai F30 S-TWIN). Further, the selected area electron diffraction (SAED) pattern was analyzed. For FT-IR analysis, the biosynthesized AgNps synthesized were air-dried at room temperature. All the measurements were carried out in the range of 500–4000 cm^–1^ at a resolution of 4 cm^–1^. The spectrum was recorded using a Cary 630 FT-IR instrument (Agilent, India). Different modes of vibrations were recognized and assigned to verify different functional groups present in the AgNPs extracted by *Colletotrichum* sp. DM16.3. All the data were corrected with the background spectrum.

For X-ray diffraction (XRD) analysis of AgNPs, a thin film of sample solution was spread evenly on an XRD grid and dried by using a vacuum dryer. XRD patterns were obtained on Shimadzu PS 7000 instrument operated at 40 kV and 30 mA with Cu Kα1 radiation (λ 1.54443). The diffracted intensities were recorded from 2 to 80° 2θ angles. The thin film on silicon cover glass (1 mm × 1 mm × 1 mm) containing the dried AgNPs sample was analyzed using Atomic Force Microscope, followed by visualization under AFM (N6520B series 6000, Agilent, United States). The thermal behavior of biosynthesized AgNPs was carried out using Netzsch STA 409 thermal analysis system (Selb, Germany). About ∼8 mg of dried AgNPs sample was loaded on the platinum pan and heated under nitrogen atmosphere at 10°C min^–1^ in the range of 20–1000°C. The analyses were performed under a gradual increase in temperature by plotting the weight percentage and heat flow against temperature.

### Thrombin Inhibition Assay and *in silico* Studies

The antithrombin assay uses a synthetic substrate of thrombin, a tripeptide linked to an inactive fluorophore (AMC), called thrombin substrate III and was conducted as described in the literature (Chan and Don, 2013). Briefly, the aqueous crude AgNPs sample (5, 10, 25, 50, 100, 200 μg/mL) was incubated with Tris buffer, pH 7.5 in a black 96-well plate. Then thrombin substrate III was added (0.2 mM) followed by the addition of thrombin (1 U/mL). Ninety-six-well plate was read at 450 nm of emission and 390 nm of excitation in a fluorimeter (SpectraMax M5e, Molecular Devices, Inc., United States) (Neethu et al., 2018). The thrombin inhibition was calculated as the decrease in fluorescence by AgNPs compared to the control fluorescence.

For *in silico* studies, the X-ray crystallographic structure of thrombin protein (PDB: 1PPB) (Bode et al., 1989) was used for docking with Ag and fungal AgNP. The AgNP structure was provided by Dr. M. Bagheri of Tehran University of Medical Sciences (Kyrychenko et al., 2015). The AgNP structure for docking was prepared in the discovery studio visualizer to remove polyvinylpyrrolidone. Docking of the Ag and AgNP was done using PATCHDOCK server (Schneidman-Duhovny et al., 2005). All the molecular dynamics simulation for the interaction of Ag and AgNP with thrombin were performed using MDWeb server (Adam et al., 2012). The GROMACS full step was used for solvation and energy minimization followed by MD trajectory generation through GROMACS-NTP at 300K for 0.5 ns.

### Thrombin Generation Assay

In this assay, the capacity of AgNPs hat inhibits the thrombin generated from plasma was assessed. The 5-weeks-old specific-pathogen free rat was acclimatized for 1 week before conducting experiments. The animal handling was carried out in accordance with the guidelines of the Committee for the Purpose of Control and Supervision of Experiments on Animals (CPCSEA). All the procedures considered were in accordance with the Animal Care and Use Committee of the National Institute of Food and Drug Safety Evaluation (NIFDS). The protocol was approved by the Institutional Animal Ethics Committees of Indian Institute of Science, Bangalore, India. Briefly, rat blood was collected via the abdominal aorta using a vacutainer tube containing sodium citrate and centrifuged at 5000 × *g* for 10 min to collect plasma. The same assay conditions of the thrombin inhibition mentioned above (see section “Characterization of Silver Nanoparticles”) were followed, except that 200 μL of rat plasma was used instead of Tris buffer and 7 μL (0.045 U/mL) of thrombin was added. The thrombin added in this assay acted as an agonist to promote the generation of thrombin from rat plasma.

### Biofilm Inhibition Assays

The effect of AgNPs was determined on the biofilm formation by both Gram-positive (*B. cereus*) and Gram-negative (*V. cholerae*) bacterial pathogens. Biofilm formation was carried out using a crystal violet assay in a 96-well polystyrene microliter plate using the modified method described earlier (Ezealisiji et al., 2017). Briefly, individual wells of the sterile microliter plate were filled with 160 μL of LB broth and inoculated with 20 μL of individual overnight grown bacterial suspension (0.6 OD at 600 nm). To the mixture, 20 μL of AgNPs solution at different concentration (0.25, 0.5, 1, 2, 5, and 10%) was added. The microliter plate was incubated for 24 h at 37°C. After incubation, the medium was discarded and gently washed with phosphate buffer saline (pH 7.2). A volume of 200 μL of a crystal violet dye (0.1%, w/v) was inoculated to each well and left for 15 min at the ambient temperature of 25°C. Then, the excess stain was rinsed off by thorough washing with sterilized Millipore water and plates were kept for drying. After drying, 200 μL of 95% (v/v) ethanol was added to the wells. The absorbance at 620 nm was measured on an ELISA reader, and values obtained were considered as an index of bacteria adhering to the surface of well wall for developing biofilms. All the experiments were performed in triplicates. The percentage of biofilm inhibition was calculated using the equation:

$$\mathrm{Biofilm}\mathrm{inhibition}\left(\%\right)=100-[{\text{OD}}_{620}\mathrm{of}\mathrm{cells}\mathrm{treated}\mathrm{with}$$

$$AgNPs\times 100)/{\text{OD}}_{620}\mathrm{of}\mathrm{non}-\mathrm{AgNPs}\mathrm{control}]$$

### Cytotoxicity Assay

The cytotoxicity of fungal AgNPs was evaluated by the cell viability assay [MTT [3-(4,5-dimethylthiazol-2-yl)-2,5-diphenyltetrazolium bromide] carried on HeLa (human cervical carcinoma) cell line as described earlier (Das et al., 2018). Briefly, cells were grown in Dulbecco’s Modified Eagle’s medium (DMEM) supplemented with 10% FBS (fetal bovine serum) in a CO_2_ incubator at 37°C for 24 h under 5% CO_2_. Penicillin and streptomycin each of 100 μg/mL concentration, respectively were added to the medium to prevent microbial growth. After the incubation period, the cells grown in 96-well flat-bottom plates were treated with different concentrations of AgNPs ranging between 5 and 100 μg/mL. Then, 10 μL of 5 mg/mL MTT solution in PBS was added to each well and incubated for 2 h at 37°C. The plate was then read at 570 nm in an ELISA reader. The results were expressed as the percentage of cell viability against the control without treatment of AgNP. The percentage of 50% of cell viability (IC_50_) was calculated using the equation:

Cellviability(%)=

$$\left({\text{OD}}_{570}\,\mathrm{of}\,\mathrm{AgNPs}\,\mathrm{treated}\,\mathrm{cells}/{\text{OD}}_{570}\,\mathrm{untreated}\,\mathrm{cells}\right)\times 100$$

### Phytotoxicity Assessment

The toxicity assessment of AgNPs was conducted by phytotoxicity assay based on the seed germination and root elongation of selected crop seeds using the method discussed by Chandankere et al. (2014), with slight modifications. *Cucumis sativus* (cucumber) and *V. radiata* (Mung bean) crop seeds were selected as bio-indicators. In brief, ten seeds were dipped in 5% Sodium hypochlorite solution for 30 min to ensure seed surface sterility and then soaked with AgNPs solution overnight. The seeds soaked in normal tap water were treated as control. Five milliliter of AgNPs solution was dispensed into a sterilized Petri-plate with Whatman filter paper No. 1 and the treated seeds were kept on the filter paper. Finally, Petri-plates were covered and incubated at room temperature for 5 days. Thereafter, the germination percentage and root elongation were estimated. Distilled water and AgNO_3_ were used as positive and negative control, respectively. Finally, the germination index was calculated as:

$$\text{GI}\left(\%\right)=\left[\left({\text{Sg}}_{\text{NP}}/{\text{Sg}}_{\text{c}}\right)\times \left({\text{Re}}_{\text{NP}}/{\text{Re}}_{\text{c}}\right)\right]\times 100,$$

where GI is the germination index; Sg_NP_ and Sg_c_ are the seed germinated in AgNPs treated and control, respectively; Re_NP_ and Re_c_ are the root elongation in AgNPs treated and control, respectively.

## Results and Discussion

### Isolation and Identification of Endophytic Fungus

**FIGURE 1:**
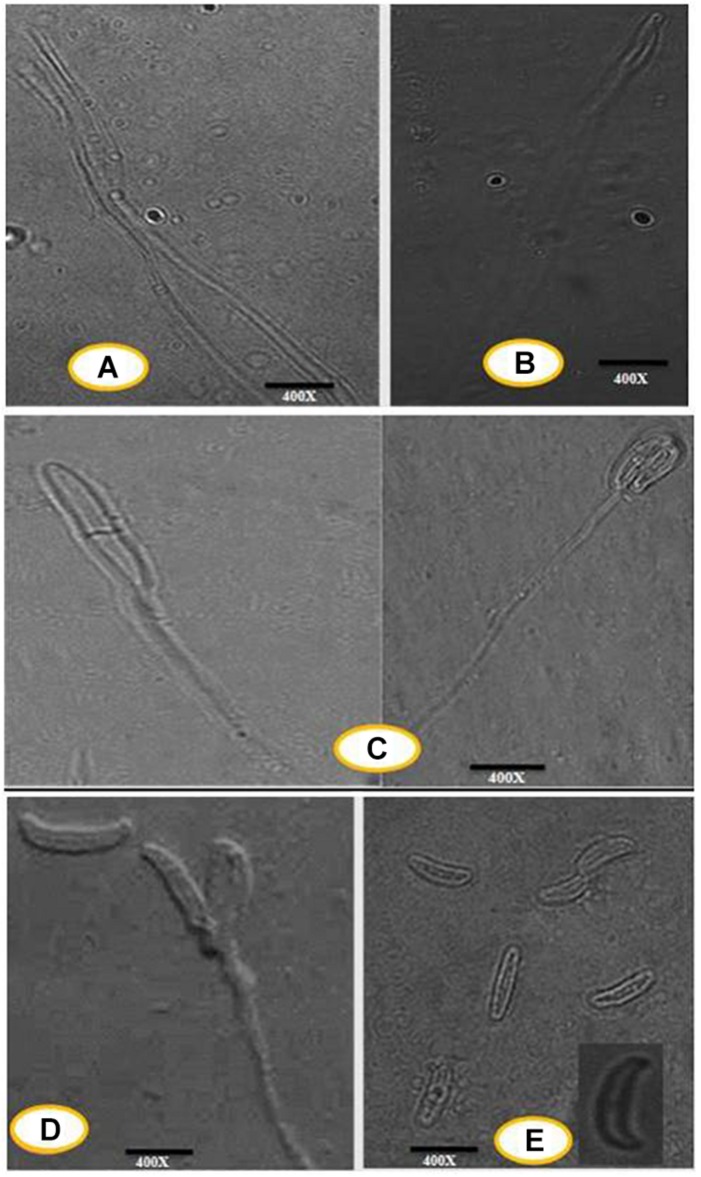
**(A)** Progressive proliferation of conidiogenous cells developments X400. **(B)** Progressive proliferation accumulation of wall layers may eventually plug the opening X400. **(C–D)** Polyphialides (more than one conidiogenous locus) X400. **(E)** Young conidia, mature conidia (insert) X400.

An efficient AgNPs producing endophytic fungal isolate, designated as DM16.3, was successfully isolated from the leaf segment of *D. metel*. Morphological identification of DM16.3 was done by colony characteristic examination. DM16.3 is opaque and smooth with black to green color, raised filamentous fungus as observed on the PDA plate (Figure 1A). Figures 1A–E represents the reproductive structures of isolated fungal strain DM16.3. The size of fusoid young conidia was in the range of 3–9 × 1.1–2.0 μm, produced in chains or single long lateral phialides. Macroconidia were fusoid and sickle-shaped with 1one or 2two septa, and 22–35 × 2.1–3.3 μm in size. Supplementary Figure S1 represents the agarose gel electrophoresis image showing partial rDNA ITS sequence (∼250 bp) from DM16.3 by genomic PCR. The result of BLASTn showed that the isolate DM16.3 was highly homologous to species from class Sordariomycetes and phylum Sordariomycete. The ITS region of rDNA sequences of DM16.3 showed 99% similarity to *Colletotrichum incarnatum* and sequence analyzed with NCBI GenBank accession number KT358850. In the present paper, the fungal strain DM16.3 is designated as *C. incarnatum* DM16.3.

### Characterization of Silver Nanoparticles

#### UV-Visible Spectral Analysis

**FIGURE 2:**
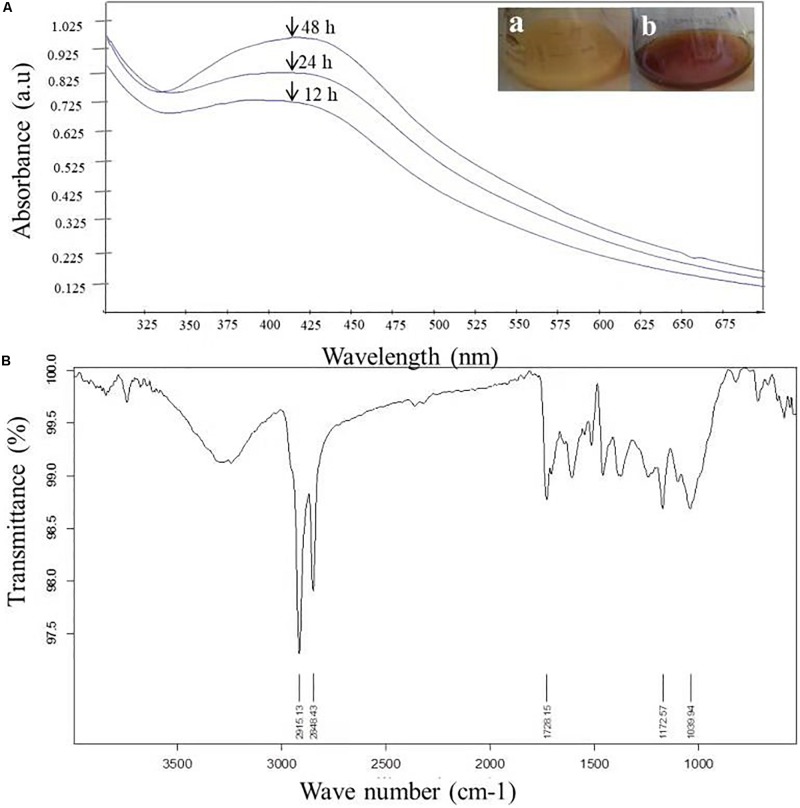
**(A)** UV-Vis spectra of AgNPs from *C. incarnatum* DM16.3 at different times. **(B)** FTIR spectrum of biosynthesized AgNPs (Inset (a) Erlenmeyer flask with *C. incarnatum* DM16.3 before exposure and (b) after exposure to AgNO_3_ ions). **(B)** FTIR spectrum of biosynthesized AgNPs.

The very first visual observation of biogenic synthesized NP is color change. On the exposure to 1 mM of silver ion solution, the initial yellow colored CF of *C. incarnatum* DM16.3 turned into dark brown color solution, while the control flask remained unchanged after the 48 h incubation period. The color change of cell filtrate was probably due to the surface plasmon vibrations of the synthesized NPs. Figure 2A represents the UV-visible spectra recorded from the *C. incarnatum* DM16.3 reaction flask at different time intervals. The characteristic surface plasmon absorption peaks were recorded at 420 nm, which further confirmed the formation of AgNPs. These observations are in agreement with what was reported in an earlier study (Chan and Don, 2013).

#### Fourier Transform Infrared Analysis

Figure 2B depicts the FTIR spectrum of the freeze-dried powder of AgNPs. It was carried out to discover the potential contact among silver and biomaterial, which could be responsible for the synthesis of AgNPs. The results demonstrated five distinct peaks at 1172, 1728, 2848, 2915, and 3285 cm^–1^. The peaks at 1728 and 3285 cm^–1^ corresponds to the binding vibration of amide I and II groups of proteins, respectively, with N-H stretching. The peaks at 2915 and 1172 could represent the methylene group and carboxylic group (C-O stretching) of the proteins, respectively (Balaji et al., 2009). These results support the presence of protein on the surface of the NPs synthesized by *C. incarnatum* DM16.3. Our finding was in concurrence with Nithya and Ragunathan (2012), who reported that proteins present in the fungal filtrate can act as the capping agent for bio-reduction and stabilization of AgNPs.

#### X-Ray Diffraction Analysis

**FIGURE 3:**
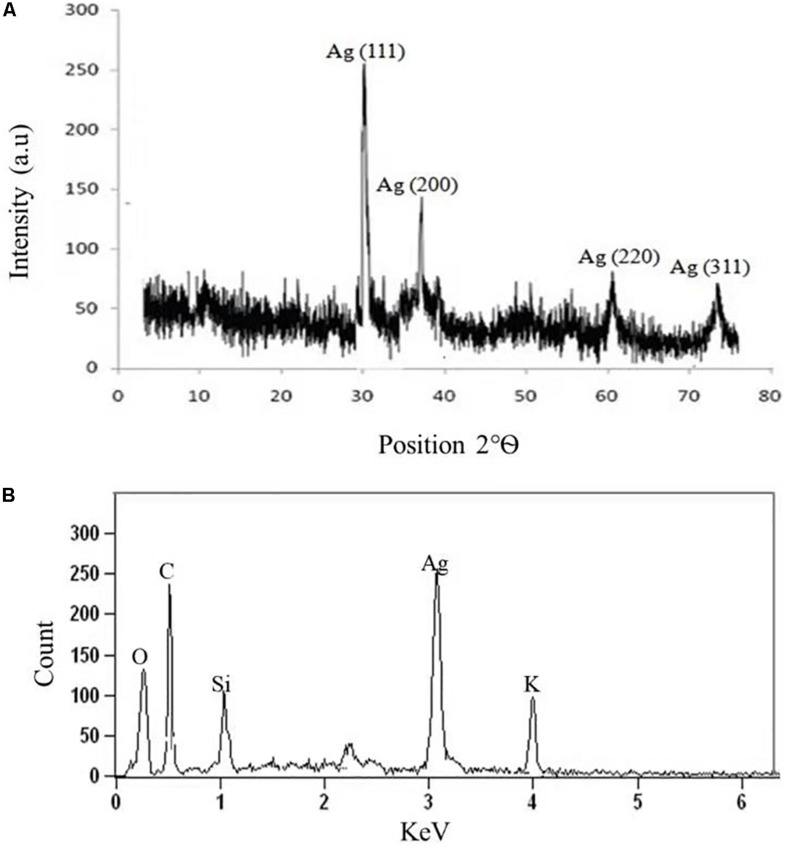
**(A)** X-ray diffraction. **(B)** Energy dispersive X-ray spectra of biosynthesized AgNPs from *C. incarnatum* DM16.3.

The crystalline nature of AgNPs synthesized by *C. incarnatum* DM16.3 was studied with XRD analysis. Intense XRD peaks at 2θ values of 32.01, 38.29, 63.21, and 75.48° assigned to the planes of (111), (200), (220), and (311), respectively, were obtained ranging from 10 to 80° (Figure 3A). The values concur well with those reported for silver (face centric cubic) by the Joint Committee on Powder Diffraction Standards file no. 04-0783, indicating the crystalline nature of Ag. Many researchers have also reported the crystalline nature of AgNPs using various fungal endophytes (Balaji et al., 2009; Baker et al., 2015).

A representative EDX pattern (Figure 3B) confirms the presence of Ag in support of XRD results. These results clearly showed the crystalline nature of AgNPs formed by endophytic fungus *C. incarnatum* DM16.3 (Bhainsa and D’Souza, 2006). A strong optical absorption band in the silver region at approximately 3 eV revealed the presence of elemental silver along with the C and O signatures that might be from the stabilizing proteins presented as capping agents on the surface of AgNPs. Liang et al. (2017) reported the silver peak at 3 eV, which is typical for the absorption of AgNPs due to surface plasmon resonance confirming the presence of nanocrystalline elemental silver.

#### Transmission Electron Microscopy Analysis

**FIGURE 4:**
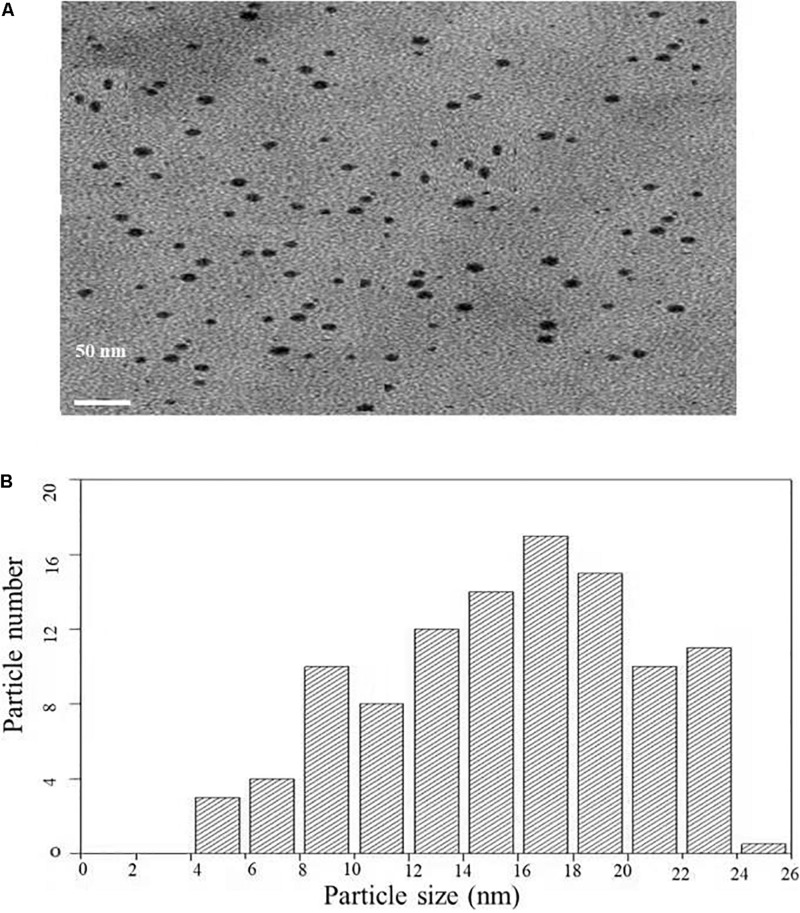
**(A)** Transmission electron microscopic image of AgNPs biosynthesized from *C. incarnatum* DM16.3. **(B)** Histogram analysis of the particle size distribution.

Transmission electron microscopy analysis was carried out to determine the size and morphology of biosynthesized AgNPs. Figure 4A represents the TEM micrograph of well dispersed and spherical shaped AgNPs. The morphology of AgNPs is predominantly spherical in size ranging from 5 to 25 nm as observed in particle size histogram (Figure 4B). However, Balaji et al. (2009) reported the synthesis of AgNPs by *Fusarium oxysporum* which was found to be hexagonal and irregular in shape.

**FIGURE 5:**
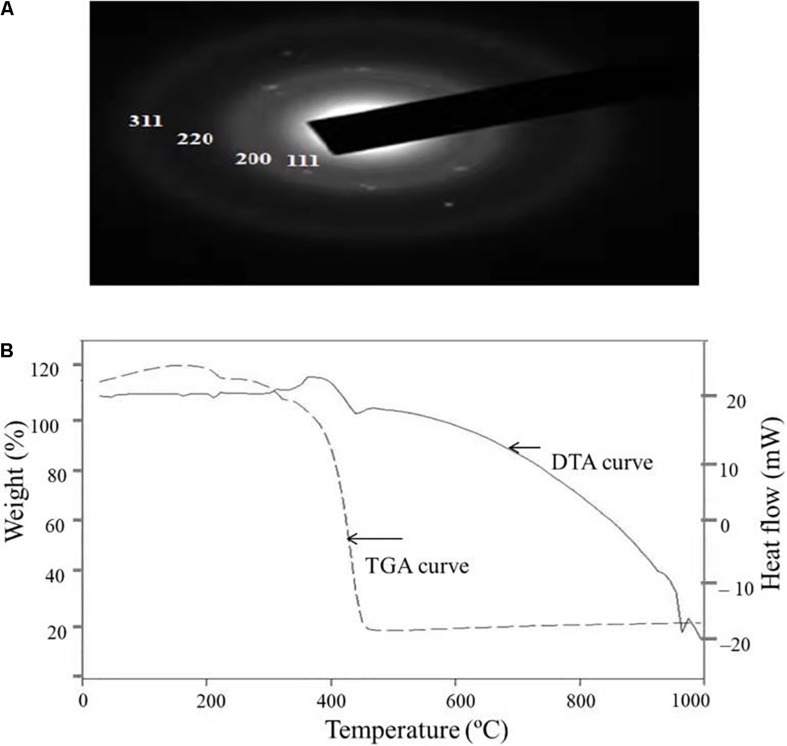
**(A)** SAED patterns of the AgNPs. **(B)** TGA-DTA thermogram of biosynthesized AgNPs from *C. incarnatum* DM16.3.

Figure 5A depicts the SAED pattern obtained from AgNps. The SAED image exhibited four bright diffraction rings arising due to reflection from (111), (200), (220), and (311) planes of face centric cubic (fcc) silver which was supported by XRD results. These results confirm the nanocrystalline nature of the AgNPs synthesized by *C. incarnatum* DM16.3.

#### Thermal Properties and Atomic Force Microscopy Analysis

Thermal properties of any NP are considered as the important attributes for various industrial applications. Therefore, the thermal behavior of biosynthesized AgNPs was studied thermogravimetric (TG) and differential thermal (DT) analysis and is depicted in Figure 5B. Apart from the gradual change in weight, the TG curve exhibited a drastic weight loss between 340 and 420°C. It was observed that the AgNPs was thermally stable up to 420°C after the weight loss of 13.75%, beyond which no further weight loss occurred. The same was established by the exothermic peak observed at 420 in the DT curve which mainly attributed to the crystallinity of AgNPs. The results showed that complete thermal decomposition and crystallization of the sample occurred simultaneously (Xiaowen et al., 2019).

**FIGURE 6:**
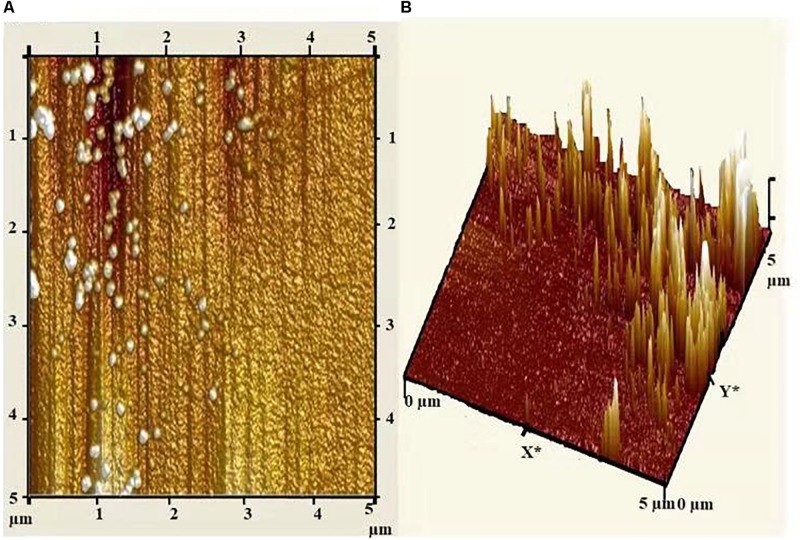
**(A)** Atomic force micrograph, and **(B)** topographical image of biosynthesized AgNPs from *C. incarnatum* DM16.3.

Atomic force microscopy (AFM) analysis of biosynthesized AgNPs showed insight into the size, shape, and distribution of NPs. Figure 6A illustrated that the AgNPs were dispersed uniformly with an average particle size of 5–25 nm, which was in concurrence with TEM and XRD results. Furthermore, the topography of the picture demonstrated the three-dimensional structure of the NPs which is depicted in Figure 6B.

### Biological Effect of Biosynthesized AgNPs

#### Thrombin Inhibition and Generation Assay

**FIGURE 7:**
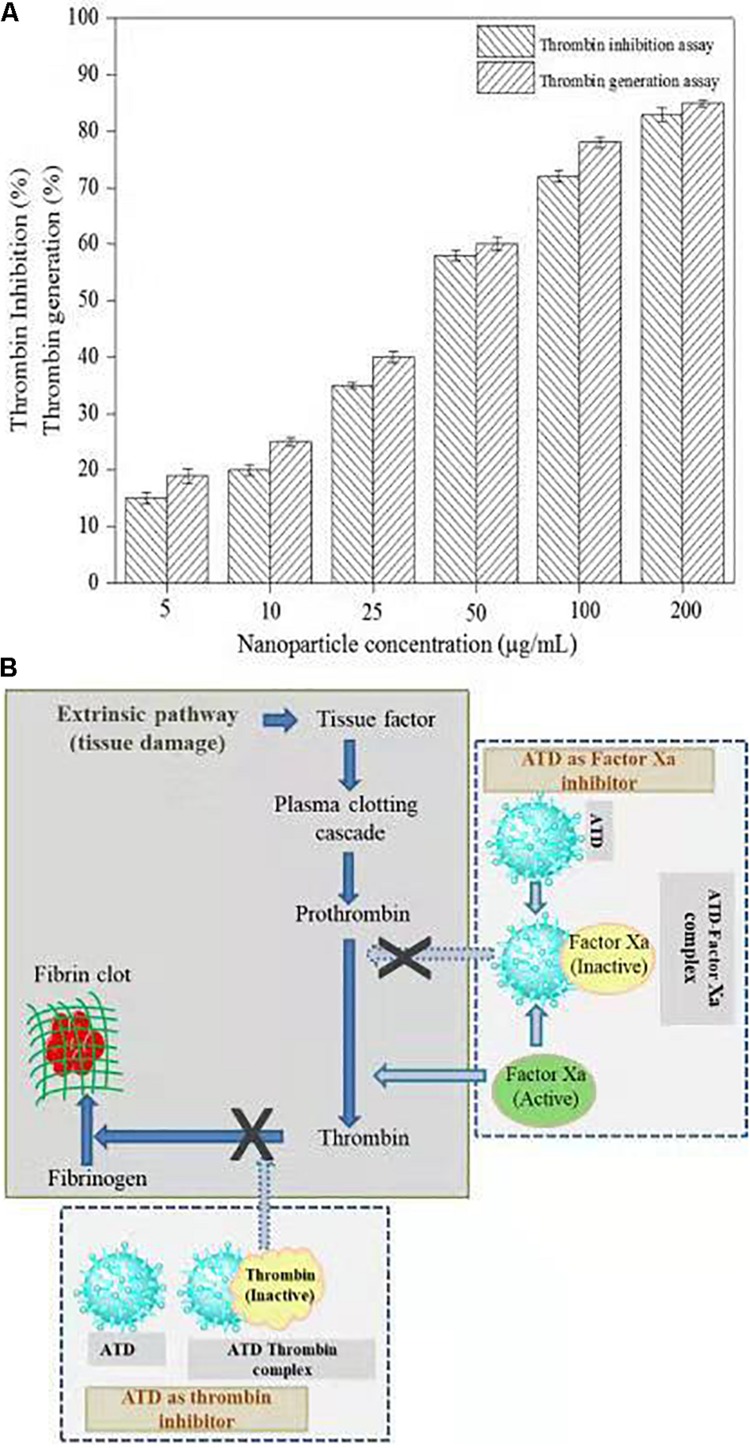
**(A)** Thrombin Inhibitory assay and thrombin generation assay of biosynthesized AgNPs from *C. incarnatum* DM16.3. **(B)** Schematic representation showing the mode of action of AgNPs as antithrobotic drug (ATD) in blood coagulation cascade. (a) ATD as a factor Xa (blood coagulating factor) inhibitor. Binding of ATD to Xa neutralizes and inhibits the coagulation factor Xa. Henceforth, reduced conversion of prothrombin to thrombin, and fibrinogen to fibrin in a sequential order (b), which eventually inhibit the blood clotting process.

Thrombin is a serine protease that regulated hemostasis responsible in the formation of obstructive blood clots, also known as thrombosis, that is a life-threatening condition related to many diseases such as stroke and cancer-associated thrombosis (Kuriakose et al., 2013). Henceforth, in the field of biomedical, there is an immense interest to develop an antithrombotic agent. In this context, AgNPs extracted from *C. incarnatum* DM16.3 was tested for thrombin inhibition capability. The AgNPs were incubated with the thrombin substrate at 5–200 μg/mL concentrations for 5 min, followed by the addition of thrombin (1 U/mL). With the treatment of AgNPs solution, there was 16, 21, 37, 56, 74, and 84% inhibition of thrombin activity at 5, 10, 25, 50, 100, and 200 μg/mL, respectively (Figure 7A). This result suggests that the synthesized AgNPs can exert thrombin inhibitory effect in a concentration-dependent manner. This, to the best of our knowledge, is the first report on the thrombin inhibitory effect of AgNPs synthesized from an endophytic fungus, *C. incarnatum* DM16.3. Earlier, Kuriakose et al. (2013) have reported similar thrombin inhibitory activity for leaf and flower extract of *C. roseus*. Nevertheless, its effect on endogenous thrombin generation potential has not been studied. Further in our studies, AgNPs inhibition of thrombin generation was carried out using rat plasma, which interestingly showed that AgNPs (200 μg/mL) could result in about 80% inhibition of thrombin generation activity. This result was concurrent with thrombin inhibition assay, where AgNPs exhibited 84% inhibition of thrombin activity. The authors have proposed the mode of action of AgNPs as an ATP and Figure 7B explicit the schematic representation showing the mode of action of AgNPs as ATD in the blood coagulation cascade.

**FIGURE 8:**
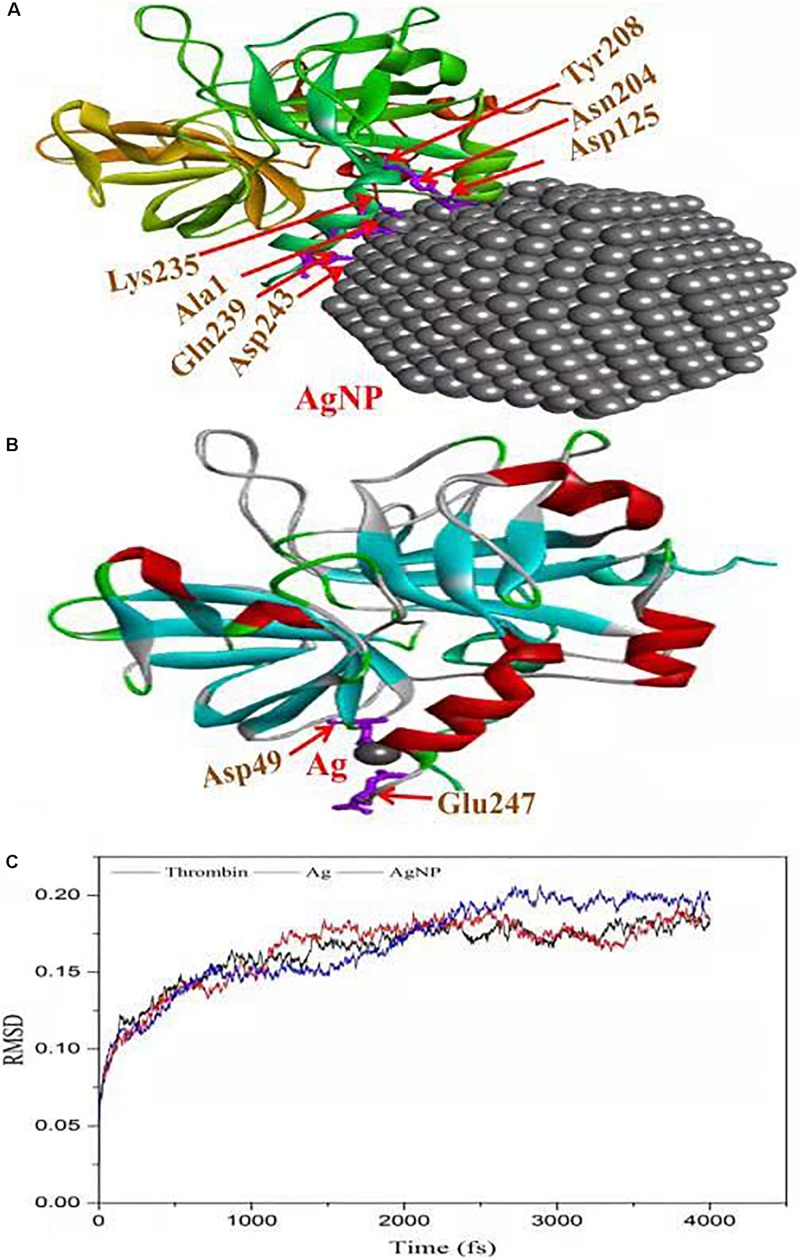
Molecular docking of thrombin protein with AgNP and single Ag atom; **(A)** Orientation of AgNP and thrombin juxtaposed towards the various amino acid residues namely Asp243, Gln239, Ala1, Lys235, Asp125, ASN204 and Tyr208. **(B)** The docking model shows an interaction of Ag to thrombin through Asp49 and Glu2476. **(C)** RSDM analysis of molecular dynamics (MD) simulations for thrombin, Ag and AgNP.

The *in vitro* analysis of AgNPs revealed that it inhibited thrombin. In the next step, to assess and predict the biological interaction of AgNP with thrombin, molecular docking studies were carried out. The closest interacting amino acid residues which contribute to the binding of thrombin protein with AgNP and Ag are presented in Figures 8A,B, respectively. The docking study revealed that AgNP had interacted well with thrombin that might cause changes in the structure of the thrombin. In order to investigate the effect of Ag and AgNP on thrombin stability, molecular dynamics simulation was performed. Root mean square deviation (RMSD) was monitored to analyze structural stability over time. The average RMSD of thrombin, thrombin-Ag, and thrombin-AgNP complex were 0.1632, 0.1641, and 0.1679, respectively. In contrast to RMSD, thrombin and thrombin-Ag complex attained equilibrium state at 3000 fs, whereas thrombin-AgNP complex needed 0.15 ns to attain equilibrium (Figure 8C). Our MD simulation analysis revealed that AgNP binding had caused thrombin unstable, which was in concurrence with the *in vitro* thrombin inhibition analysis.

#### Cytotoxicity Assay

**FIGURE 9:**
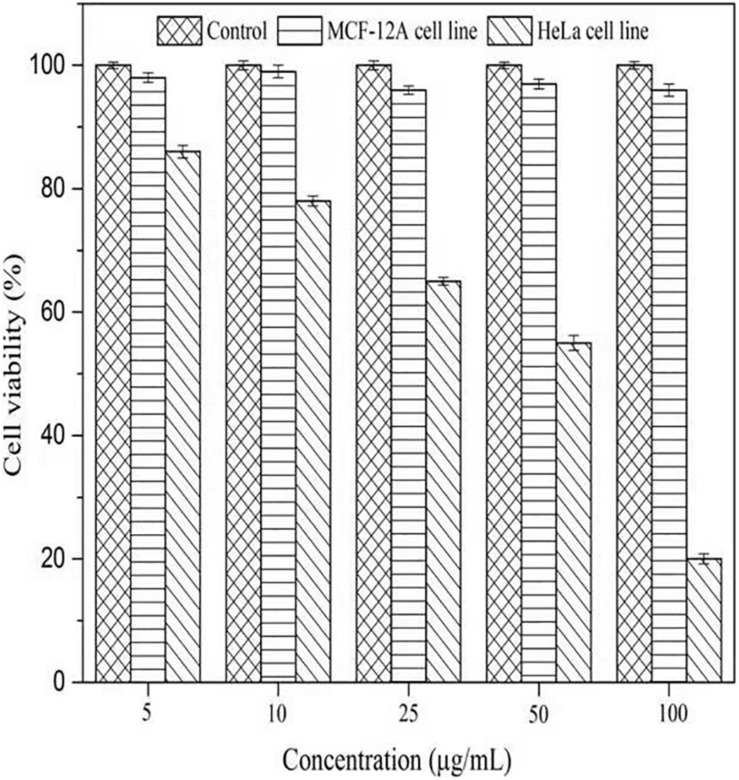
Cell viability assay of AgNPs against normal cells (MCF-12A human breast epithelial cell line) and cancerous cells (HeLa human cervical carcinoma cell line).

To evaluate the cytotoxicity of AgNPs, the normal cells (MCF-12A, human breast epithelial cell line) and cancerous cells (HeLa, human cervical carcinoma cell line) were treated with varying concentrations of AgNPs (5, 10, 25, 50, and 100 μg/mL) for 24 h. The biosynthesized AgNPs were found to have negligible toxicity and the viability of MCF-12A cells was slightly affected up to 100 μg/mL (Figure 9). The cell viability of MCF-12A was reduced to 6.73% at 100 μg/mL of AgNPs. Due to the negligible toxicity of AgNPs against normal cell lines, *C. incarnatum* DM16.3 derived AgNP can be effectively used as an anticancer agent. In contrast, the viability was reduced in a dose-dependent manner in HeLa cell lines and the cytotoxicity of the AgNPs was observed with the inhibitory concentration (IC_50_) value of AgNPs was 50 μg/mL. The cytotoxic effects of AgNPs might be due to the interference with the proper functioning of cellular proteins that thereafter provoked apoptosis by the mitochondrial pathway in HeLa cells (Liang et al., 2017). A significant reduction in the HeLa cell lines viability was observed at a higher concentration of AgNPs. The cell viability was reduced to 80.29% at 100 μg/mL. The cell viability was reduced by 6.73% in the case of MCF-12A and 80.29% for HeLa cell line at 100 μg/mL concentration. Xiaowen et al. (2019) showed IC50 value to be 500 μg/mL of AgNPs against human breast carcinoma cell line (MDA-MB-231). But the effective doses used in these studies were considerably higher than those observed in the present study (IC_50_, 50 μg/mL).

#### Antibiofilm Potential

The bacteria have the ability to form an organic polymer matrix referred as “biofilms” on the surface, which is involved in key problems associated with the medicine and food industry. According to Singh et al. (2013), AgNPs have a significant effect on microbial adhesion and biofilm disruption, which has become an effective method to diminish biofilm formation and combat colonization by pathogenic microorganisms. In the present study, extracted AgNPs were tested for biofilm inhibition potential against *B. cereus* (Gram-positive) and *V. cholerae* (Gram-negative) having known for their ability to form a biofilm. Table 1 explicit the inhibition of bacterial biofilm by different concentrations of AgNPs synthesized by *C. incarnatum* DM16.3. Test organisms were grown in 96 μL plate wells with different concentrations (0.25–10 μg/mL) of AgNPs to form biofilm for 24 h. In both cases, the amount of biofilm formation was decreased by an increase in AgNPs concentration. The results illustrate that the activity of AgNPs is highest at the concentration of 10 μg/mL, with the inhibition rate of 85.1% (IC_50_, 2 μg/mL) and ∼ 46.5% against *Vibrio* sp. and *Bacillus* sp., respectively. From the results, it was observed that the AgNPs were better antibiofilm agents against the Gram-negative bacteria. This difference in the inhibitory activity of AgNPs might be due to the presence of an external layer with negative charges in Gram-negative bacteria that is attracted by the weak positive charge of the AgNPs (Dakal et al., 2016). In contrast, the Gram-positive bacteria presents a thick layer that reduces the binding of AgNPs (Magdi and Bhushan, 2005). Our results were in concurrence with the literature which reports significant inhibitory activities against Gram-negative bacteria (Xiaowen et al., 2019).

**TABLE 1:** Inhibition of bacterial biofilm by different concentration of AgNPs synthesized by *C. incarnatum* DM16.3.

**Microorganism**	**Concentration of AgNPs (μg/mL)**	**Biofilm inhibition (%)**
*Vibrio cholerae*	0.25	29.5 ± 0.5
	0.5	37.2 ± 0.8
	1	45.1 ± 0.2
	2	58.6 ± 0.9
	5	69.0 ± 0.5
	10	85.1 ± 0.2
*Bacillus cereus*	0.25	15.1 ± 0.2
	0.5	23.9 ± 0.6
	1	29.3 ± 0.1
	2	34.7 ± 0.9
	5	40.1 ± 0.7
	10	45.6 ± 0.2

#### Phytotoxic Activity

**FIGURE 10:**
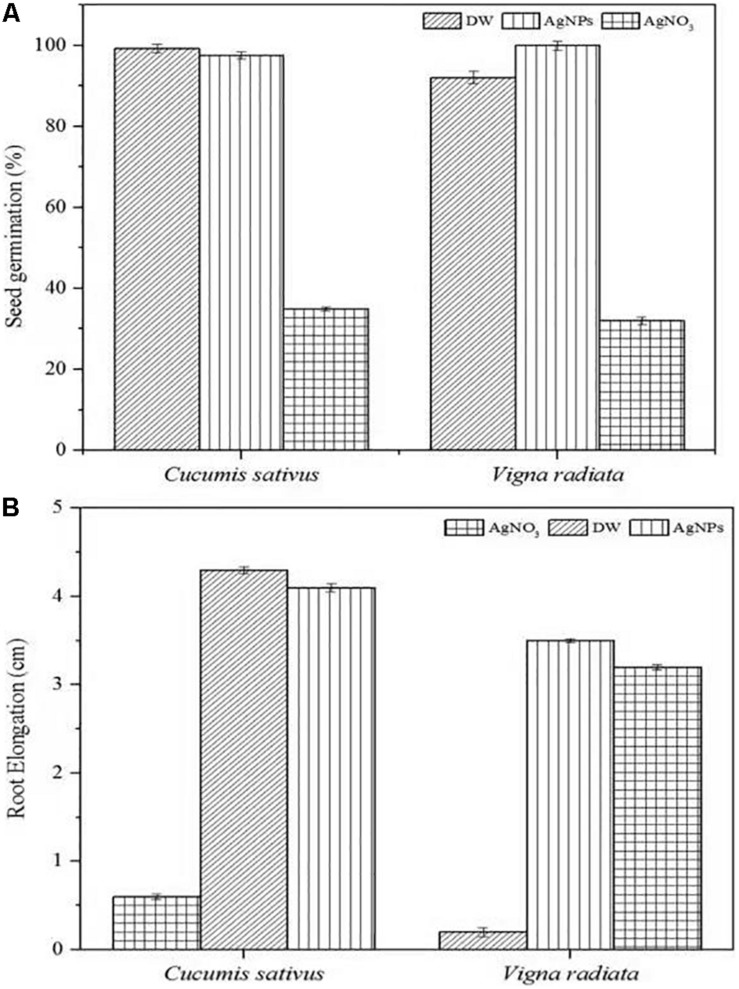
Phytoxicity activities of *C. incarnatum* DM16.3 derived-AgNPs. **(A)** Seed germination percentage when treated with AgNPs, distilled water (positive control) and AgNO_3_ (Negative control), and **(B)** Normalized root elongation when treated with AgNPs, distilled water (positive control) and AgNO_3_ (negative control).

The phytotoxic analyses of AgNPs were tested on the seeds of *C. sativus* and *V. radiata*. The phytotoxicity assay performed was based on seed germination and root elongation. The results revealed that the AgNPs obtained from *C. incarnatum* DM16.3 did not show any inhibitory effect on seed germination and root elongation against both the tested seeds when compared with the control that exhibited inhibitory characteristics. However, the highest seed germination (%) and root elongation were observed in *C. sativus* with 99.4% and 4.0 cm, whereas the lowest seed germination (%) and root elongation was obtained against AgNO_3_ with 28.5% and 0.4 cm, respectively (Figures 10A,B). Interestingly, leaves and secondary root growth was noticed in both the tested seeds. Our observation was in agreement with Yin et al. (2012) who reported a non-appearance of phytotoxicity of the AgNPs against *Lolium multiflorum*. A growth index of 80% has been used as a bio-indicator to confirm the absence of phytotoxicity of the AgNPs (Jiang et al., 2018).

## Conclusion

This study has shown that the endophytic fungus *C. incarnatum* DM16.3 isolated from the leaves of *D. metel* is a promising strain for the biosynthesis of AgNPs. Extensive characterization of the synthesized AgNPs revealed that these NPs were spherical in shape with a size ranged from 5 to 25 nm. The composition and crystalline nature of AgNPs was confirmed by FTIR, XRD, EDS, and SAED analyses. This is the first report on the role of AgNPs been extracted by endophytic fungus *C. incarnatum* as a thrombin inhibiting agent. The *in silico* docking revealed that the AgNPs had interacted well with thrombin that might cause changes in the structure of the thrombin, further inhibited thrombin. The biosynthesized AgNPs were further tested for the antibiofilm activities and exhibited significant biofilm inhibition activities against Gram-negative bacterium. AgNPs also reported noteworthy cytotoxic by inhibiting the HeLa cell lines and non-toxic effect against normal cell lines. Furthermore, AgNPs have not exhibited any phytotoxic activities on seeds of crops claiming its safety to the environment. Thus, we conclude that the biosynthesized AgNPs may be used as a promising and eco-friendly drug agent in biomedical applications. Nevertheless, further research works, particularly *in vivo* studies should be carried out before AgNPs could be considered for biological application.

## Data Availability Statement

All datasets generated for this study are included in the article/Supplementary Material.

## Ethics Statement

The animal handling was carried out in accordance with the guidelines of the Committee for the Purpose of Control and Supervision of Experiments on Animals (CPCSEA). All the procedures considered were in accordance with the Animal Care and Use Committee of the National Institute of Food and Drug Safety Evaluation (NIFDS).

## Author Contributions

RC and JC designed the experiment and analyzed the data. RC and HZ wrote the manuscript. RC, VS, and AP conducted the experiment. YS contributed to the resources access. HZ and XQ contributed to the data interpretation and commented on the manuscript. XQ conceived and supervised the overall project. All authors listed have contributed to manuscript revision, read, and approved the submitted version.

## Conflict of Interest

The authors declare that the research was conducted in the absence of any commercial or financial relationships that could be construed as a potential conflict of interest.
