# Supplementary material for: Pleiotropic Functions and Biological Potentials of Silver Nanoparticles Synthesized by an Endophytic Fungus
Source: Front Bioeng Biotechnol. 2020 Feb 21;8:95. doi: 10.3389/fbioe.2020.00095 (PMC7047737; doi:10.3389/fbioe.2020.00095)
Supplement: Supplementary file 1 [file Table_1.doc]

**Pleiotropic Functions and Biological Potentials of Silver Nanoparticles Synthesized by an Endophytic-Fungus**

Chandankere Radhika^1,2^, Chelliah Jayabaskaran^2^, Subban Kamalraj^2^, Shanadrahalli C. Vanitha^2^, Parvez Amreesh^1^, Hossain M. Zabed^1^, Sharma C. Yogesh^3^, Xianghui Qi^1*^


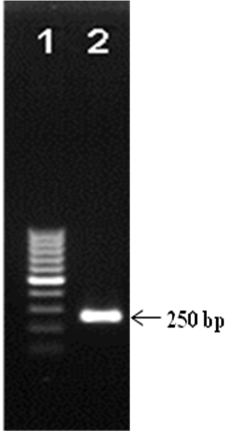


**Figure S1.** Agarose gel electrophoresis showing partial rDNA ITS sequence (~250bp) of ***C.*** *incarnatum* DM16.3 isolated from *Datura metel*. Lane 1: 100bp DNA marker, Lane 2: endophytic fungus showing ~250-bp
